# Rotational Atherectomy, Orbital Atherectomy, and Intravascular Lithotripsy Comparison for Calcified Coronary Lesions

**DOI:** 10.3390/jcm12237246

**Published:** 2023-11-23

**Authors:** Kamila Florek, Elżbieta Bartoszewska, Szymon Biegała, Oliwia Klimek, Bernadeta Malcharczyk, Piotr Kübler

**Affiliations:** 1 Students’ Scientific Group of Invasive Cardiology, Institute of Heart Diseases, Wroclaw Medical University, 50-369 Wroclaw, Poland; bartoszewska.ela@gmail.com (E.B.); szymonbiegala02@gmail.com (S.B.); rybkajr11@gmail.com (O.K.); bernadeta.malcharczyk@gmail.com (B.M.); 2 Institute of Heart Diseases, Wroclaw University Hospital, 50-556 Wroclaw, Poland; pkubler75@gmail.com; 3 Department of Cardiology, Faculty of Medicine, Institute of Heart Diseases, Wroclaw Medical University, 50-367 Wroclaw, Poland

**Keywords:** rotational atherectomy, orbital atherectomy, intravascular lithotripsy, coronary artery calcifications, percutaneous coronary interventions, IVUS, OCT

## Abstract

In order to improve the percutaneous treatment of coronary artery calcifications (CAC) before stent implantation, methods such as rotational atherectomy (RA), orbital atherectomy (OA), and coronary intravascular lithotripsy (IVL) were invented. These techniques use different mechanisms of action and therefore have various short- and long-term outcomes. IVL employs sonic waves to modify CAC, whereas RA and OA use a rapidly rotating burr or crown. These methods have specific advantages and limitations, regarding their cost-efficiency, the movement of the device, their usefulness given the individual anatomy of both the lesion and the vessel, and the risk of specified complications. This study reviews the key findings of peer-reviewed articles available on Google Scholar with the keywords RA, OA, and IVL. Based on the collected data, successful stent delivery was assessed as 97.7% for OA, 92.4% for IVL, and 92.5% for RA, and 30-day prevalence of MACE (Major Adverse Cardiac Events) in OA—10.4%, IVL—7.2%, and RA—5%. There were no significant differences in the 1-year MACE. Compared to RA, OA and IVL are cost-effective approaches, but this is substantially dependent on the reimbursement system of the particular country. There is no standard method of CAC modification; therefore, a tailor-made approach is required.

## 1. Introduction

Coronary artery calcification (CAC) is a rising issue among the modern population. The prevalence of this problem is linked to many factors, such as the older age of the patients; increased morbidity of diabetes mellitus, chronic kidney disease, dyslipidemia, and hypertension; cigarette smoking; and others [1]. It is a reliable predictor of death caused by myocardial infarction and coronary artery disease (CAD), which remain the frontmost reasons for mortality and loss of Disability-Adjusted Life Years (DALYs) globally. The majority of such deaths take place in low- and middle-income countries, accounting for nearly 7 million deaths and 129 million DALYs annually. Moreover, patients who survive myocardial infarction have at least a five- to six-fold higher annual mortality rate, compared to those who are not afflicted with CAD [2,3,4].

**Figure 1:**
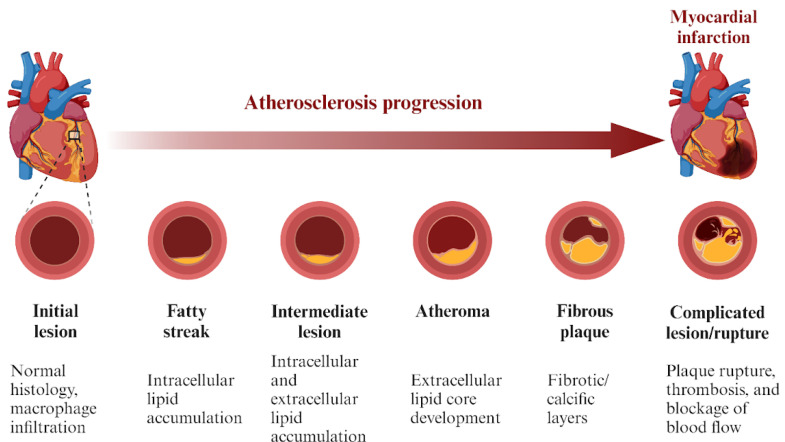
The progression of atherosclerosis [6].

CAC is a pathological process, associated with the progression of advanced atherosclerosis (Figure 1), that may imply the existence of CAD. The process initiates in the necrotic core of the atherosclerotic plaque. The precursory part of CAC is associated with the death of the macrophages and smooth muscle cells, as well as the remaining necrotic debris, serving as nucleating sites for calcium phosphate crystal formation. Moreover, the matrix vesicles and apoptotic bodies released during the death of the inflammatory cells provide the scaffolding for calcific lesions. The process is associated with reduced local expression of mineralization inhibitors and with a loss of control over the local concentration of calcium and phosphate. Microcalcifications are most commonly formed in areas where there is a local decrease in collagen fibers, where they subsequently aggregate into greater and tougher masses [5]. Coronary artery calcification can be divided into two types, based on whether it occurs in the vascular medial layer, or within the intima of the vascular wall, the latter being the most common type of CAC [6].

Calcium deposits in the arterial wall obstruct vascular blood flow, and the rupture of these plaques is often followed by thrombotic vessel occlusion, which represents the main mechanism of myocardial infarction [5]. Various methods of percutaneous coronary intervention have been developed in order to restore optimal blood flow and regain recanalization. However, in some severe cases, high-risk and complex PCI may need to be performed, which, without special tools, may result in lower success rates of the procedure. The term ‘high-risk PCI’ is linked to complex coronary artery disease, such as multivessel or left main coronary artery disease, and with hemodynamic compromise, such as shock or highly depressed function of the left ventricle. Moreover, it can be used to describe a procedure on a patient with clinical comorbidities such as advanced age, heart failure, peripheral vascular disease, diabetes mellitus, or previous cardiac surgery. The definition of complex PCI, on the other hand, is associated with complex, anatomic coronary lesions which might be characterized by severe calcification, extreme tortuosity or length, extensive thrombotic burden, or chronic total occlusion. The location of such lesions can occur at a coronary bifurcation or in a degenerated saphenous venous bypass graft [7].

Some of the most challenging coronary lesions are associated with chronic total occlusions (CTOs). CTO refers to the occlusion of a coronary artery, related to luminal discontinuity with a duration greater than or equal to 3 months. Such obstruction is composed of atherosclerotic plaque and homogeneous or composite thrombotic components. With time, the lesions become more severe and more difficult to cross and remove due to modulation by fibrous tissue and the calcification process. Furthermore, inability to insert the guidewire beyond such an occlusion remains the most common cause of PCI failure in CTO. In terms of procedural success, the rate of successful modification of CTO-combined lesions stands at 68%, whereas that of non-CTO lesions lies at 95% [8,9].

Consequently, PCI does not exude its full therapeutic effect in vessels containing lesions, which are complex and moderately or severely calcified [10]. Calcified plaques in coronary arteries obstruct balloon dilatation and effective stent delivery, leading to malposition, stent underexpansion, and drug-eluting polymer coat degradation. This is an independent risk factor for recanalization failure [11]. In cases where the risk of mortality, cardiac death, or nonfatal myocardial infarction cannot be reduced using PCI, the optimal medical treatment is preferable [12].

In order to avoid poor procedural outcomes and an increased risk of significant adverse cardiovascular events, procedures like debulking the plaques before stent implantation have been implemented. Nowadays, the emphasis has changed from simply facilitating stent administration in difficult-to-cross lesions to lesion preparation, in order to enhance and maximize stent expansion [11]. The most common procedure is lesion modification, which would ease the administration and expansion of drug-eluting stents (DES) [10].

New methods were created to optimize stent deployment, ranging from rotational atherectomy (RA), created in the 1980s, to orbital atherectomy (OA), with the first human use reported in 2014, and coronary intravascular lithotripsy (IVL), whose use was described for the first time in 2017. RA may seem like an old technique when looking at the date of its first human use; however, it was initially rejected and later improved to develop the atherectomy which we know today [11,13,14,15].

This study aimed to analyze the differences between the three methods of CAC modification before stent implantation. The aforementioned procedures vary in many aspects. Notably, the size of the treated vessel and the severity of calcification differ, which determines the choice of a particular procedure. Other important variables include risks during the procedure, complications, operability, size of the debris, as well as costs [16,17]. The method of work is also diversified: atherectomy uses a burr or a crown to remove plaques and lithotripsy uses sound waves to crack the calcium [16,17]. For uncrossable lesions, RA or OA are recommended, while for suboptimal balloon expansion in bigger vessels, IVL is preferable [18].

However, the methods can be combined. The suboptimal effect of one method usually leads to using two of them together, for example, in the case of a failed IVL, an orbital-tripsy (OA and IVL) can be performed [19]. New combinations are also applicable to severely calcified in-stent restenosis. Neo-atherosclerosis can be treated using atherectomy techniques and IVL, because of its synergistic calcium modification effect [20].

## 2. Mechanism of Action

Atherectomy techniques, both rotational and orbital, use a rapidly rotating burr or a crown to modify calcified plaques [17].

**Figure 2:**
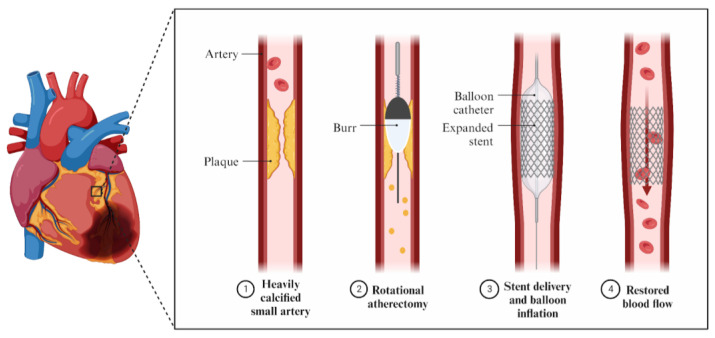
Mechanism of action of RA.

The RA device is called a rotablator and contains a diamond-encrusted elliptical burr (Figure 2). It rotates at high speeds using a helical driveshaft moving over a guidewire. The speed usually ranges from 140,000 to 160,000 rpm, but in severe cases, a speed even more than 190,000 rpm can be used. The device pulverizes the calcified lesions into tiny particles that can pass through the bloodstream. The maximum burr-to-artery ratio should range from 0.4 to 0.6 [21]. RA is a good choice for tight and heavily calcified lesions. However, the rotablator can only cut the plaque forward and it is possible for its burr to become stuck. Therefore, it should be kept running until withdrawn [16]. Nowadays, RA is implemented under various circumstances, such as high-risk patients and complex anatomy [22,23]. Fundamental elements of the optimal RA technique include short ablation passages from 15 to 20 s [21]. Prolonged passages are associated with higher heat generation and a higher possibility of artery perforation, microembolization, or vessel dissection [24]. In addition, the recommendation to avoid decelerations above 5000 rpm is also included in the North American Expert Review of Rotational Atherectomy [21]. Burr sizes usually vary from 1.25 to 2.00 mm; however, producers can supply the catheterization laboratories with burrs in sizes 0.35–2.5 mm [25]. Furthermore, in RA, the use of heparin, RotaGlide lubricant, and vasodilators is recommended, as it helps to prevent vasospasms, lower generated heat, and no-/slow-flow complications [21,26]. According to the standard protocol for rotational atherectomy, it is possible to temporarily avoid a pacemaker by using atropine to prevent heart blocks. It is also acceptable to use smaller burrs and lower the speed in order to reduce the chances of those incidents [27].

**Figure 3:**
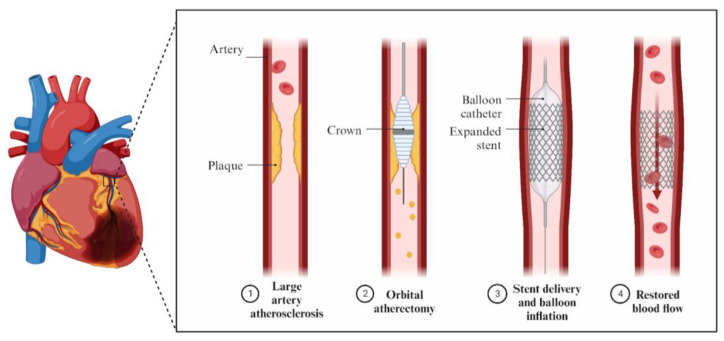
Mechanism of action of OA.

Conversely, OA uses a crown which rotates, but in orbital motion (Figure 3). It can work at two speeds—80,000 rpm or 120,000 rpm—and the treatment for each lesion should start at the lower speed [28]. Similarly to the rotablator, this device mechanically crushes the blockage created by the calcified plaque. There are two types of the OA system that differ in the shape of the diamond-coated distal tip of the crown, called classic and Micro Crown, respectively [29]. The size of the crown is 1.25 mm for both devices, but the tip of the Micro Crown allows for easier traversing through a channel with a smaller diameter. Temporal vessel occlusion is not associated with OA, in contrast to RA and IVL. Moreover, orbital movement enables continuous blood flow during lesion modification, better microparticle flush, and lower heat generation [30]. The advantage of OA is its ability to ablate forward and backward, which may be helpful in tortuous and aorto-ostial lesions and may help to avoid burr entrapment. It is a good choice for debulking larger vessels because it creates more calcium modification in plaque situated in the artery with a larger lumen. It can also modify noncalcified lesions [16]. Studies show a longer procedural time of OA when compared to RA [31]. However, a greater duration of the procedure in OA may be associated with the best practice recommendations, which include a rest time longer than each treatment interval. The maximum passage time is recommended to be 30 s and the device emits a sound when the passage time is 25 s [32]. In contrast to RA, there are no specific recommendations in terms of flush; however, the infusion of ViperSlide alone is sufficient. Otherwise, if additional drug use is necessary, it is preferable not to include vasodilators in the ViperSlide and instead use intracoronary nitroglycerin between runs [30,32]. The temporal pacemaker placement is recommended in the instructions for the Diamondback 360^®^ Coronary Orbital Atherectomy System as it should be implemented while treating the lesions in the right coronary artery (RCA) or dominant circumflex lesions. However, a retrospective multicenter analysis showed that the activation of the pacemaker in OA was significantly lower than in the RA group. Moreover, pacing in OA was needed only in 0.9% of all patients evaluated in the previously mentioned study [33].

**Figure 4:**
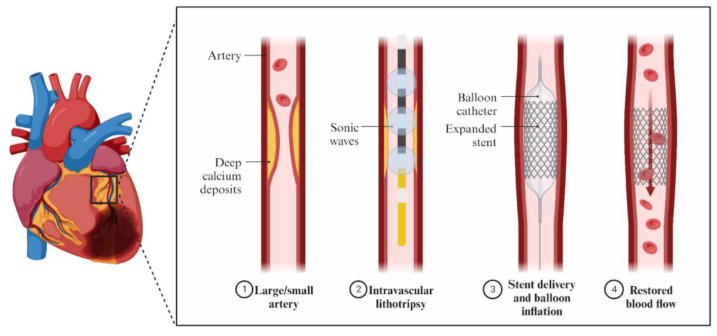
Mechanism of action of IVL.

The IVL catheter is a balloon-based device with lithotripsy emitters producing uniformly distributed sonic waves (Figure 4). They cause intraplaque calcium fractures [34]. The advantage of IVL is its ability to modify deep calcium, which is impossible with the use of RA and less likely with OA [35]. The standard technique includes fitting the device to the size of the reference artery 1:1 and delivering the catheter to the target by mono-railing over a guidewire. IVL usage is independent of the treated artery lumen size, contrary to RA and OA. An IVL balloon is then inflated to achieve 4 atm in the lesion and 10 pulses are transmitted. Afterward, it is inflated temporarily to 6 atm and deflated to allow blood flow. These steps are usually repeated to achieve patency in the artery; however, temporal vessel occlusion is present. The device can deliver a total of 80 or 120 impulses [18]. The benefits of a catheter-like delivery include reduced injury of the interior layer of the vascular wall and a low complication rate [34]. IVL is a useful tool in the modification of suboptimal stent expansion and was proven to be safe and efficient in increasing the lumen of underexpanded stents [36]. IVL is not an appropriate choice for tight stenoses. However, thanks to balloon inflation, IVL does not suffer from wire bias like RA and OA [37]. Lastly, although there is a learning curve in IVL, it is not steep because device delivery is similar to standard catheter-based PCI. The ability to perform IVL procedures among cardiologists may be acquired faster, compared to atherectomies [38]. Side-branch protection with a guidewire can be safely achieved using IVL, without the potential risks of wire entrapment or breakage that may occur with RA or OA [39]. No arrhythmias were recorded during IVL procedures [40].

## 3. Intravascular Imaging in RA, OA, and IVL

Calcified coronary lesions pose a number of issues for coronary angioplasty, such as suboptimal acute PCI outcomes leading to more frequent late stent failure [41]. In order to correctly assess when atherectomy is required, the methods of intravascular imaging were invented. These techniques measure parameters such as the amount of calcium, arc, and wall thickness, which can be subsequently used as predictors for adequate stent expansion [42]. Although not applied prior every time OA, RA, or IVL is performed, intravascular ultrasound (IVUS) imaging and OCT are proven to enhance the clinical outcomes of individuals receiving percutaneous coronary intervention (PCI) and have been associated with a lower in-hospital mortality rate [35,41,43,44].

**Figure 5:**
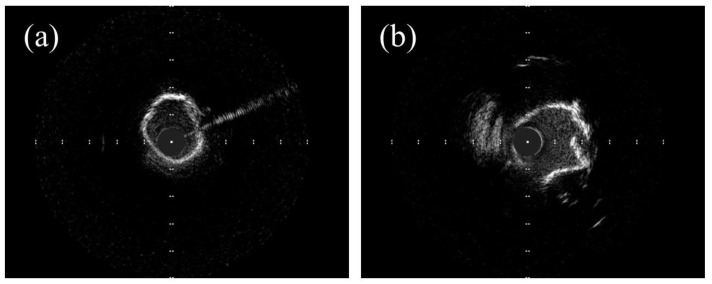
IVUS images of (**a**) calcified artery and (**b**) artery after calcium plaque debulking.

IVUS imaging (Figure 5) is important in the identification, therapy guiding, and post-treatment evaluation of coronary artery disease. IVUS provides real-time cross-sectional images that allow the operator to precisely measure the vessel wall morphology, the vessel lumen opening, and other associated blood and vascular parameters by cannulating a tiny ultrasound transducer-attached catheter into an artery using a different ultrasound frequency, which varies from 40–45 MHz to 50–60 MHz [45]. As the primary component of an IVUS system, the ultrasound transducer is critical in determining the IVUS imaging performance [46]. 

**Figure 6:**
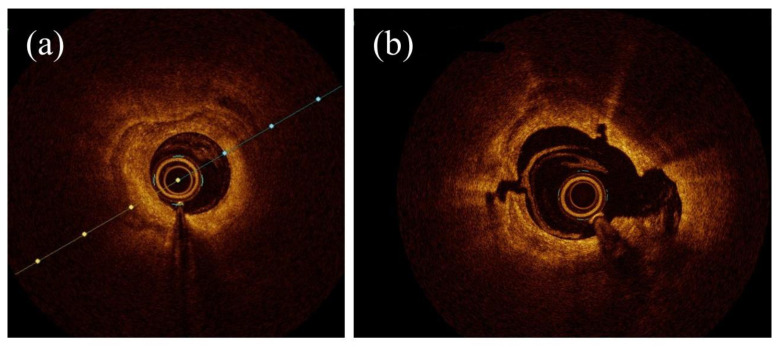
OCT images of (**a**) calcified artery; (**b**) artery after calcium plaque debulking.

OCT is an intravascular imaging technique that produces high-quality images of the morphological components of the vessel’s wall, safely and effectively (Figure 6). OCT is now a widely used intravascular technique for studying coronary arteries, stent placement, and arterial injury [47]. This non-invasive technology for cross-sectional tissue imaging commonly employs light in the near-infrared spectral range, which penetrates tissue to a depth of several hundred microns. The backscattered light is detected using an interferometric setup to rebuild the sample’s depth profile at a chosen position. Cross-sectional images of the tissue structure can be acquired using a scanning OCT beam [48].

Although similar, the techniques use different wave sources: IVUS imaging is based on ultrasound (40–45 MHz or 50–60 MHz) and OCT on near-infrared light. IVUS has a wider axial resolution—it allows for a complete vessel wall visualization—and greater penetration depth in soft tissue than OCT. They have a comparable maximum pullback length. IVUS allows for aorto-ostial lesion visualization and plaque burden at the lesion site. OCT enables the evaluation of cross-sectional calcium not only from different angles but also to assess its thickness. In terms of lipidic plaque assessment, IVUS comes in handy with attenuated plaques and OCT can be used in the measurement of lipidic plaque and cap thickness. In pre-intervention assessment, OCT is superior in determining calcium thickness. Both OCT and IVUS are useful for assessing stent expansion following PCI. In situations such as tissue protrusion through the strut, stent malposition, and stent edge dissection, OCT provides a better morphological evaluation. Moreover, during follow-up visits, OCT can provide a better diagnostic insight in cases such as old stent expansion, tissue coverage, and neo-atherosclerosis, whereas IVUS allows for a positive remodeling of vessel wall detection [45]. The choice of intravascular imaging technique is a part of the operator’s choice-making process, and if it is needed, both techniques can be used. 

Intravascular imaging, such as IVUS or OCT, is an essential tool when there is a need to explore complex anatomical structures. The left main coronary stem has a different structure from the other segments. This causes a significant challenge during PCI and is the reason for poorer clinical outcomes. Studies also show a greater need for revascularization compared to other lesions [49]. Another issue concerns coronary bifurcation lesions, which are the stenosis of the coronary artery near the origin of a major side branch. They occur in 15–20% of performed PCI, and because of traditional angiography’s limitations, are associated with poorer procedural success rates and a greater risk of adverse events [50]. The use of intravascular imaging helps to properly guide the PCI device (Table 1) and ensures immediate improvement of the procedure, as well as providing better long-term outcomes [49].

### 3.1. OCT Assessment in RA, OA, and IVL 

As OCT allows for a more detailed morphological insight into calcified lesions compared to IVUS, it is important to highlight the significant differences it makes in the results of RA, OA, and IVL. The study comparing RA to OA showed significantly greater stent expansion in the RA group (99.5% compared to 90.6% in OA), a bigger percentage of lumen area increase (72.2% in RA compared to 39.2% in OA), and a larger maximum atherectomy area in RA (1.34 vs. 0.83 mm^2^) [35]. On the other hand, another study showed significant differences in the depth of dissections. OA was related to deeper tissue modifications (1.14 vs. 0.82 mm) and had a lower percentage of stent malposition than RA [52].

The second study compared RA and IVL and showed a significantly higher number of fractures in IVL, and the fractures caused by IVL were longer compared to those caused by RA. As a result, the total volume of the fractures was larger with IVL. However, RA was associated with bigger acute lumen gain [53]. Another study found that the minimal stent area was similar after both RA and IVL. There were also no differences in stent symmetry or strut malposition [54].

As demonstrated, each method has its advantages and disadvantages. No method is considered superior to others based on the OCT results. However, those imaging techniques may be helpful in assessing which modification tool would be more appropriate for specific calcium distribution and lesion morphology.

### 3.2. OCT vs. IVUS in RA, OA, and IVL

In all of the techniques considered in this review, IVUS is used most commonly [55]. However, it is important to examine the issue of whether, according to particular techniques, OCT-guided or IVUS-guided procedures are associated with better stent expansion or clinical outcomes.

OCT-guided RA procedures for the treatment of calcified coronary artery lesions resulted in significantly greater stent expansion compared to IVUS-guided RA [56,57,58]. There is a lack of data on OA and comparisons between the utility of particular intravascular imaging tools; however, some data is provided by an ECLIPSE study, which includes an OCT-guided OA group [59]. On the other hand, OCT was the method of choice in the Disrupt CAD III study regarding IVL procedures [60]. However, an in vivo study of the sensitivity assessment of 440 calcified lesions shows that OCT detected calcium in 76.8% and IVUS in 82.7% of lesions, whereas angiography detected calcium in only 40.2% of lesions [61].

## 4. Effectiveness

According to research, there were two randomized trials carried out with DES implantation, which provided detailed data about the outcomes of RA: an older study—the ROTAXUS (Rotational Atherectomy Prior to Taxus Stent Treatment for Complex Native Coronary Artery Disease) study—and a newer study—the PREPARE–CALC study (Comparison of Strategies to Prepare Severely Calcified Coronary Lesions). The number of patients included in those RA studies was 240 and 200, respectively [62,63]. The ROTAXUS trial assessed the procedural success of RA as 92.5%, clinical success as 91.9%, and 30-day MACE as 5%. In PREPARE-CALC, the trial RA success, defined as a successful stent delivery and expansion, was noted in 98% of procedures [63]. A higher procedural success for RA was observed in PREPARE-CALC, even though the inclusion criteria incorporated patients with more advanced CAD compared to ROTAXUS. The PREPARE-CALC trial included patients with severe calcifications, as well as those with left main trunk (LMT) stenosis, whereas the ROTAXUS trial considered patients with moderate and severe calcified lesions and excluded those with LMT calcifications [62,63]. However, it is difficult to compare those results without assessing the impact of operators’ experience, the influence of the learning curve, the use of newer materials, and a better understanding of the factors predicting stent failure as those studies were carried out during different periods of time [64]. The important difference that may be suspected to have an influence on procedure effectiveness, besides those previously mentioned, is the use of optical coherence tomography (OCT) in the PREPARE-CALC study. OCT helped operators with the correct decision-making process before and after DES implantation. The differences between those randomized trials are also visible in the follow-up data. In the ROTAXUS study, MACE were defined as a composite of death, new myocardial infarction (MI), and target vessel revascularization (TVR) at 9 months and was assessed as 24.2%. In contrast, the 9-month MACE rate including the same events as in the PREPARE-CALC trial can be scored as 7% [62,63].

The ORBIT II trial assessed the OA system as a safe and efficient method with an efficacy endpoint of 88.9%. Moreover, high rates of successful stent delivery (97.7%), as well as low rates of in-hospital complications (each reported <1%), were noted [65]. In this study, MACE were defined as the occurrence of acute MI, stroke, perforation, dissection, or thrombus. The 30-day MACE were assessed as 10.4%, and the 12-month MACE as 16.4%. This study was conducted in 2014 and included 443 patients; however, more randomized trials regarding OA are needed. A currently ongoing ECLIPSE randomized trial may suggest a strategy for OA usage [59]. Interestingly, OA has been shown to decrease procedure failure and reintervention rates compared to RA [11]. Although there are no data available from newer randomized trials, it is possible to gain insight from a meta-analysis comparing the RA and OA in-hospital outcomes. Data from eight observational studies comparing RA and OA were analyzed. Significant differences between those techniques were reported, in line with more frequent coronary dissections and perforations in OA and a higher rate of long-term MACE (1-year), long-term TVR, and in-hospital as well as 30-day MI in RA [60].

With regard to IVL, the Disrupt CAD I-IV studies were performed uniformly in 12 countries and enrolled 628 patients. IVL’s procedural success was defined as successful stent delivery with residual stenosis < 50% using core laboratory assessment without in-hospital MACE established, and was concluded in those trials to be 92.4%. MACE were defined as the occurrence of clinical events such as cardiac death, MI, or TVR and 30-day MACE were assessed as 7.2% [60]. In CAD III and IV trials’ 1-year MACE were, respectively, 13.8% and 9.4% [60,66]. The Disrupt CAD III study had an OCT sub-study, whose 1-year results have not been analyzed yet.

A comparison of CAD III trials and ORBIT II is possible due to the similar inclusion criteria, definitions, and composite endpoints. In both studies, the 30-day MACE were mainly driven by non-Q-wave MI (NQWMI) [18]. However, a cross-trial comparison between other studies is unfeasible due to a different design and stent use.

There is no standard method of calcified coronary artery modification prior to DES implantation as some lesion types respond better to one device than another. Consequently, more quality randomized trials with longer follow-ups are needed [17].

## 5. Periprocedural Complications and Short-Term Outcomes

The serious clinical complications that may occur during RA include MI, stroke, death, and procedural complications: dissection, perforation, and slow-flow and no-flow phenomena, due to distal embolization. They are observed in 6–15% of patients [22,67,68]. No-flow can lead to heart failure, MI, cardiogenic shock, and death [69]. Those clinical events affect procedure effectiveness and some of them are included in the composite endpoints of the trials.

No-flow and slow-flow are angiographically confirmed to diminish the perfusion of the myocardium. They are more frequently observed in RA (6–15%) than in other methods: 0.9% in OA while no associations with no-flow were reported in IVL [30,70]. No-flow as a complication of the procedure is associated with the microcapillary embolization formed by the debris produced during ablation. From a mechanical perspective, the comparison of the particle size in RA (10–15 μm) and OA (2–3 μm) is crucial compared to the diameter of the capillaries (~8 μm) [28,71]. Means of preventing no-flow are associated with appropriate patient selection for the RA procedure, and if the risk is high, other techniques may be considered. Patients with more complex atherosclerotic lesions are suspected to be more susceptible to the no-flow phenomenon occurrence, which did not affect the 1-year mortality in these studies [72]. Nevertheless, the proper selection of burr sizes and the recommended use of flushing are important to prevent the occurrence of no-flow [4]. The majority of studies focus on the no-flow complication in the field of MI, which implies a low role of RA in these analyses. However, one study showed that the no-flow phenomenon in RA is associated with higher mortality in long-term follow-up [25]. The impairment in the blood flow due to RA is treated using intracoronary administration of verapamil, diltiazem, adenosine, nicardipine, or nitroprusside [73].

In several studies, the incidence of death after RA was 0–4% of patients, while MI occurred in 1–14%, dissection was found in 1.7–7%, and vascular perforation was reported in 0–2% [62,74,75,76,77]. Burr entrapment or rotawire interruption occurred at a frequency of less than 1%, and such complications were often associated with the need for urgent cardiac surgery [74,78,79]. The risk of those life-threatening conditions can be minimalized thanks to the prevention of the most common causes of dissections, which are balloon over-dilatation or balloon rapture. The same causes can lead to perforation; moreover, the data indicate that intravascular imaging can predict artery perforation [80]. Coronary artery perforation during PCI is most likely while advancing the guidewire. The risk of perforation is relevant while treating CTOs with the use of stiff hydrophilic wires [81,82]. It has also been confirmed that a burr-to-artery ratio of more than 1:1 increases the risk of perforation, so prevention in this area should be implemented according to the current recommendations [10,83]. The management of coronary artery perforation in hemodynamically unstable patients requires pericardiocentesis, whereas in hemodynamically stable patients, prolonged balloon inflation should be performed for 5–30 min at a low pressure of 1–2 atm and reversal of anticoagulation should be considered. If these procedures are not sufficient to close the perforation, subsequent implantation of a covered stent or coil embolization in the distal narrow parts is necessary. The last treatment option is surgical treatment [80]. According to dissections, those which do not limit the flow may not require any specific intervention. In contrast, those with artery lumen occlusion or flow restriction should be treated to keep the artery open using balloon dilatation, usually with stent implantation or, in the case of intramural hematoma, prior to implantation, a cutting balloon can be used. It is crucial to maintain the wire position in the true lumen, but if the position is lost, some CTO techniques are available to regain it [84].

A meta-analysis including seven studies comparing the short-term outcomes of RA and OA revealed that RA is associated with a higher risk of in-hospital and 30-day MI, but a lower prevalence of coronary artery dissection and perforation. Additionally, RA was associated with a longer procedural fluoroscopy time [85,86].

Severe complications are very rare after IVL [70,87]. IVL is considered an atraumatic technique, which is in contrast to RA or OA. In studies conducted on a large group of patients undergoing lithotripsy treatment on coronary vessels, 14 cases of MACE were observed among 308 patients [14,70,87,88,89]. In addition, six cases of mild vascular dissection (type A–C) were found in this group. Severe dissection (type D–F) was not observed. Vascular dissection may be caused by the rupture of a lithotripsy balloon using too high a pressure under unfavorable vascular conditions [90].

In another registry with a longer follow-up analyzing the results of RA after DES implantation in a group of over 200 patients, the 9-month follow-up showed a 17.7% cumulative rate of cardiac events (death 4.4%, infarction 3.4%, revascularization of the treated vessel 9.9%, and revascularization of the treated lesion 6.8%) and only two events of late stent thrombosis [77,91,92].

## 6. Long-Term Outcomes 

The long-term outcomes are important when choosing the method of interventional treatment. In our analysis, we considered long-term outcomes as the incidence of MACE one year after percutaneous coronary intervention. MACE were defined as the occurrence of cardiac death, MI, and TVR.

The analysis comprises seven distinct studies. According to the data, there were no significant differences in the one-year outcome among individual methods. After one year, MACE occurred in an average of 15% of patients who underwent RA (with a range of 13.2% to 26% depending on the study) [76,93,94]. Similar outcomes were observed in patients who underwent OA, with a mean one-year MACE rate of 14.4% (11% to 16.4%) [94,95,96]. An insignificantly lower MACE rate was observed in patients who underwent IVL, with an average of 13.2% (ranging from 9.4% in Japan to 13.8% in the US and Europe) [60,66]. 

In terms of RA, in the Euro4C Group multicenter European study, which included 966 patients, the MACE rate was relatively low at 13.2% [93]. A study by Milad El Hajj et al. conducted at a single center reported a relatively high MACE rate of 26% [94]. It included a small number of patients and had a limited impact on the overall outcome of methods such as RA, but the results of the procedure may have depended on the hospital performing the intervention. Consequently, more trials reporting the long-term MACE after RA are needed.

### Double Antiplatlet Therapy in Complex PCI

PCI increases the risk of thrombosis. Therefore, a prescription of double antiplatelet therapy (DAPT) to lower the risk of ischemia is considered essential following the procedure [97]. DAPT is a medical treatment approach that involves the concurrent use of two antiplatelet medications to prevent the formation of blood clots and usually involves a combination of the P2Y12 inhibitor and acetylsalicylic acid. The current guidelines recommend pharmacotherapy over a period of 6 to 12 months. However, some studies indicate that shortened DAPT after complex PCI, such as one requiring atherectomy, significantly reduces the risk of major bleeding, while still maintaining the same ischemic risk and mortality rate [98]. One of the studies compared the effect of ticagrelor monotherapy with ticagrelor in combination with aspirin for 12 months in patients after an atherectomy procedure and 3 months of DAPT. The results showed that shortened DAPT or ticagrelor with a placebo resulted in a lower risk of all-cause death, myocardial infarction, or stroke [99]. This provides an interesting perspective and is a signal to expand research in this area in atherectomy patients.

## 7. Cost-Effectiveness

Economic analyses mainly compare RA to OA or IVL. There are no data comparing all three methods. Although such an assessment should be made, the available data with comparisons of other methods to the most commonly used method, RA, give us insight into the cost of the procedure, as well as the cost-effectiveness curves evaluated for each technique. The factors considered in the economic studies for the majority of devices were cost, material usage, procedure duration, radiation exposure, and reintervention rate, which implies a budget spent per patient in good health. Moreover, the procedural success and MACE rate were included in those evaluations to assess the cost-effectiveness profile of the procedures.

OA is a cost-effective approach compared to RA due to its improved performance and a lower rate of reinterventions [17,28]. An analysis carried out in Japan found that for OA devices at a local price, the technique was a more economical option with the added advantage of insight into clinical outcomes [17]. Another meta-analysis determined that the only difference between the two atherectomies was the fluoroscopy time, which was lower for OA [86]. In terms of IVL and RA, there are comparisons claiming that IVL is less expensive due to its lower overall resource use [100,101]. There are also differences between studies concerning radiation levels between IVL and RA [85,100]. It is necessary to adapt these data to specific regions, as the price of equipment can vary between them. The character of the learning curve of the procedures under consideration should be taken into account, as there is a significant difference between the learning process of atherectomy and IVL, which has an impact on the procedure’s cost-effectiveness. In addition, there should be clear guidelines on the use of intravascular imaging, as this also affects the cost of the procedure.

## 8. Conclusions

Each technique analyzed in the review has its advantages and disadvantages; therefore, there is no definitive method of calcified coronary artery modification prior to DES implantation (Table 2). An individual approach, that is, selecting the right device for a particular patient, seems to be the most sensible strategy. Intravascular imaging may help with the assessment of the morphology of the calcified lesions, and therefore assist in choosing the most beneficial technique. However, as atherectomies and IVL have different learning curves, it is highly important to adjust the technique used during the procedure to the operators’ experience and the centers’ resources. In addition, the most widely used modification technique appears to be that of the highest cost; thus, there is a need to optimize costs and consider the use of other techniques based on their cost-effectiveness profile. However, it is substantially dependent on the reimbursement system of the particular country. More quality randomized trials and meta-analyses are needed in order to develop consistent guidelines for interventional cardiologists. 

## 9. Limitations

The limitations of this study are mainly caused by the lack of direct analysis considering all three techniques in the original studies. For this reason, some comparisons between those techniques had moderately different criteria or definitions. In addition, there are not a lot of randomized trials in this field, due to the fact that there were mainly observational studies considered, which have some weaknesses.

## Figures and Tables

**Table 1:** Recommendations on the use of intracoronary imaging in clinical practice [51].

IVUS	Diagnosis of intermediate stenosis of the left coronary artery trunk
IVUS/OCT	Optimization of stent implantation procedures in native arteries
	Coronary artery recanalization procedures (guidewire position assessment, true/false lumen navigation)
	Studies on the progression/regression of atherosclerosis
IVUS > OCT	Optimizing the left coronary artery trunk angioplasty procedure
	Imaging for spontaneous coronary artery dissection
	Vasculopathy after heart transplantation
OCT > IVUS	Optimizing revascularization in patients with current coronary artery calcifications
	Intracoronary imaging for suspected acute coronary syndrome
	Diagnosis of the causes of stent implantation failure
	Diagnosis of neo-atherosclerosis

**Table 2:** RA, OA, and IVL comparison: mechanism of action, periprocedural complications, effectiveness, short-term and long-term outcomes.

Category	Parameter	RA	OA	IVL
Mechanism of action	Device	Rotating burr (140,000–160,000 rpm)	Rotating crown (80,000–120,000 rpm)	Emits sonic waves (80–120 impulses)
	Independent of lumen size	−	−	+
	Modifies deep calcium	−	+/−	+
	Temporal vessel occlusion	+	−	++
	Tight stenosis	+	+	−
	Wire bias	+	+	−
	Modifies noncalcified lesions	−	+	−
	Treatment of in-stent restenosis	−	−	+
Periprocedural complications	Wire entrapment	++	+	−
	No-/slow-flow risk	6–15%	0.9%	None reported
	Dissection	Lower risk	Higher risk	Rare
	Perforation	Lower risk	Higher risk	Rare
Effectiveness	Procedural success (stent delivery)	92.5%, 98%	97.7%	92.4%
Short-term outcomes	30-day MACE	5%	10.4%	7.2%
Long-term outcomes	1-year MACE	15%	14.4%	13.2%

“+”—applicable/present; “−”— no applicable; If more than one sign is used it is associated with feature intensity.

## Data Availability

Not applicable.
